# Comparative Proteomics Analyses of Pollination Response in Endangered Orchid Species *Dendrobium Chrysanthum*

**DOI:** 10.3390/ijms18122496

**Published:** 2017-11-23

**Authors:** Wei Wang, Hongyang Yu, Tinghai Li, Lexing Li, Guoqiang Zhang, Zhongjian Liu, Tengbo Huang, Yongxia Zhang

**Affiliations:** 1 Guangdong Provincial Key Laboratory for Plant Epigenetics, College of Life Sciences and Oceanography, Shenzhen University, Shenzhen 518060, China; wangweiszuniv@163.com (W.W.); hongyangyu@szu.edu.cn (H.Y.); thli90@163.com (T.L.); lilexing02@163.com (L.L.); tengbohuang@szu.edu.cn (T.H.); 2 Key Laboratory of Optoelectronic Devices and Systems of Ministry of Education and Guangdong Province, College of Optoelectronic Engineering, Shenzhen University, Shenzhen 518060, China; 3 Shenzhen Key Laboratory for Orchid Conservation and Utilization, the National Orchid Conservation Center of China and the Orchid Conservation & Research Center of Shenzhen, Shenzhen 518114, China; zhang2384539@163.com (G.Z.); liuzj@sinicaorchid.org (Z.L.)

**Keywords:** pollination response, proteomics, functional classification, *Dendrobium chrysanthum*

## Abstract

Pollination is a crucial stage in plant reproductive process. The self-compatibility (SC) and self-incompatibility (SI) mechanisms determined the plant genetic diversity and species survival. *D. chrysanthum* is a highly valued ornamental and traditional herbal orchid in Asia but has been declared endangered. The sexual reproduction in *D. chrysanthum* relies on the compatibility of pollination. To provide a better understanding of the mechanism of pollination, the differentially expressed proteins (DEP) between the self-pollination (SP) and cross-pollination (CP) pistil of *D. chrysanthum* were investigated using proteomic approaches—two-dimensional electrophoresis (2-DE) coupled with tandem mass spectrometry technique. A total of 54 DEP spots were identified in the two-dimensional electrophoresis (2-DE) maps between the SP and CP. Gene ontology analysis revealed an array of proteins belonging to following different functional categories: metabolic process (8.94%), response to stimulus (5.69%), biosynthetic process (4.07%), protein folding (3.25%) and transport (3.25%). Identification of these DEPs at the early response stage of pollination will hopefully provide new insights in the mechanism of pollination response and help for the conservation of the orchid species.

## 1. Introduction

The majority of flowering plants are hermaphroditic, that is their flowers often have male and female organs within close proximity on the same plant or even within the same flower. The mechanisms controlling floral pollination response, especially the self-/non-self-recognition, are of crucial importance for maintaining genetic variability and species survival [1,2].

The self-incompatibility (SI), a genetic mechanism that is widespread among flowering plants, promotes out-crossing to increase genetic diversity within plants [3,4]. SI is found in approximately 40% of flowering plant species and in at least 100 families [2,5,6]. It permits the pistil to discriminate between self-pollens and cross-pollens and further to mediate the rejection of self-pollens. In flowering plants, the stigma is the receptive surface of the pistil for pollination where the integration of numerous events occurs during pollen recognition in SI [7,8,9]. The first checkpoint of SI responses is the pollen-pistil interactions. This process leads to the acceptance of compatible pollens or the rejection of self-incompatible pollens [10,11,12,13]. Then the pollen grains start to adhere, hydrate, and germinate on the stigma surface. The pollen tubes then will grow through the pistil, using a specialized mechanism of tip growth [14,15].

There are two classic systems of SI: gametophytic SI (GSI) and sporophytic SI (SSI), based on modes of genetic control of pollen SI phenotype [16]. GSI is relatively widespread, and it has been found in the *Solanaceae*, *Papaveraceae*, *Ranunculaceae*, *Leguminosae*, *Onagraceae*, *Scrophyulariaceae*, *Rosaceae*, and *Poaceae*. In GSI, the haploid pollen determines the incompatibility, while in SSI it is determined by the the diploid genotype of its parent. The SSI has been found in *Brassicaceae*, *Asteraceae*, and *Convolvulaceae* [17].

Recent studies have made significant progresses in understanding the molecular mechanism of SI in flowering plants. In *Brassica*, the SI responses began with the interaction between stigma-specific S receptor kinase (SRK) and the pollen coat protein *S*-locus Protein 11/*S*-locus Cysteine-Rich (SP11/SCR). The binding of SP11 induced the autophosphorylation of SRK and further triggered the downstream signaling cascades resulting in the self-pollen rejection [18,19,20]. In *Petunia hybrida*, the SI system is controlled by a single *S*-locus with multiple haplotypes. The *S*-locus consists of two genes, one is the female determinant *S-RNases* expressed in pistil, and the other is *SLF/SFB* (*S*-locus F-box/*S*-haplotype-specific F-box), the male determinant, expressed in pollen [21]. In *Papaver papaveris*, the signal molecule S proteins were encoded by the *S*-locus in pistilis responsible for the SI. When pistil S proteins interact with incompatible pollen S receptors, this self-incompatible interaction triggers an intracellular Ca^2+^ signaling cascade(s) to effect rapid inhibition of pollen growth [22].

The pollination mechanisms displayed by orchid flowers has aroused many interests among biologists over the centuries, since the first comprehensive study published by Darwin [23,24,25]. Research in this field is mainly focused on the relationship among orchid species and their pollinators. However, the molecular and the genetic mechanisms underlying SI are poorly understood in the orchid family. The *Dendrobium*, one of the largest genera within the *Orchidaceae*, comprises about 1200 species [26]. Most species are distributed in Australia, tropical Asia and Australasia, and many endemic species are reported along its distribution range [27,28,29,30]. This genus consists of both Self-compatibility (SC) and Self-incompatibility (SI) species [31]. *Dendrobium chrysanthum* is one of the SI species [32]. The sequence and phylogenetic analysis of the *S-RNase* and *SLF-interacting SKP1-like1 (SSK1)* found that none of the *S-RNases* in this species are the S-determinant, but do have *SSK1* genes in *D. chrysanthum*, suggesting that a none-*S-RNase* based GSI in *D. chrysanthum* may involve diverse mechanisms which are still elusive [32]. The pollen-stigma checkpoint is the first step in the pollination process [10,11,12,13], and dissection of the pollination response at an early stage could help to understand sporophytic mechanisms in this species. Furthermore, the *D. chrysanthum* is a highly valued ornamental orchid which is also used in the preparation of traditional herbal medicines by Chinese and the Khasi tribe of India. Due to the excessive collection and habitat destruction, the status of this species has been declared endangered [33].

Proteomic analysis is a powerful tool that can provide systematic understanding of a biological event at the molecular level. In this study, we conducted a proteomics analysis on self-pollens or cross-pollens treated pistils derived from *D. chrysanthum* to investigate the early proteomic response between self-pollination and cross-pollination. Two-dimensional electrophoresis (2-DE) was performed to identify differentially expressed proteins, and characteristics of these proteins were examined by bioinformatic analysis. To the best of our knowledge, this is the first application of proteomic approaches to investigate the early responseof pollination in orchids and will hopefully help to identify genes involved in pollination process in *D. chrysanthum*.

## 2. Results and Discussion

### 2.1. Protein Profiles of D. chrysanthum in Un-Pollination, Self-Pollination and Cross-Pollination

After extracted from pistils of un-pollination, self-pollination (SP) and cross-pollination (CP) *D. chrysanthum*, the total proteins were subjected to isoelectric focusing (IEF) and sodium dodecyl sulfate polyacrylamide gel electrophoresis (SDS-PAGE). After scanning, the 2-DE maps of the SP and CP pistils were obtained. To accurately and quantitatively analyze proteomic changes, spot volume differences of more than 2-fold between two identical spots were defined as significant. Three independent repeats were conducted in order to get reliable results.

**Figure 1:**
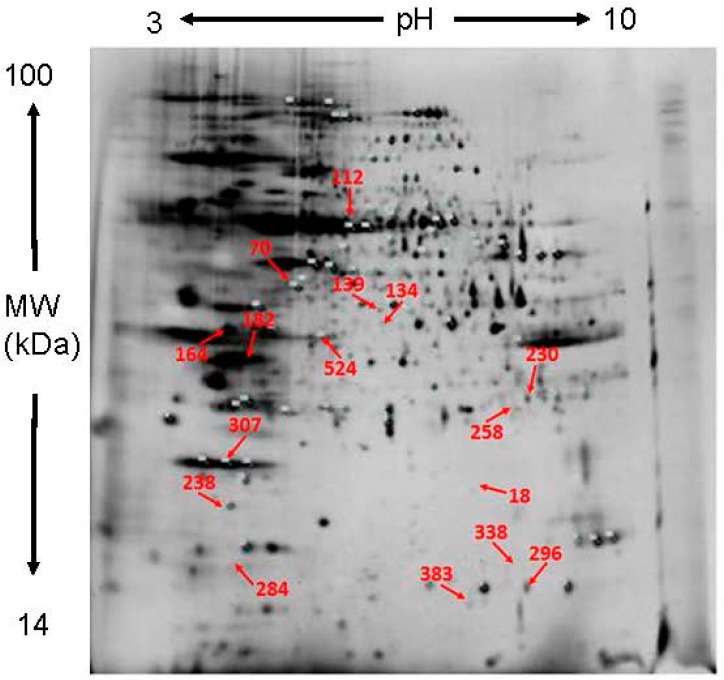
Two-dimensional gel electrophoresis (2-DE) of un-pollinated pistils. Red arrows demonstrate some selected protein spots, which were numbered and collected for identification by matrix-assisted laser desorption/ionization-mass spectrometry (MALDI-MS).

**Figure 2:**
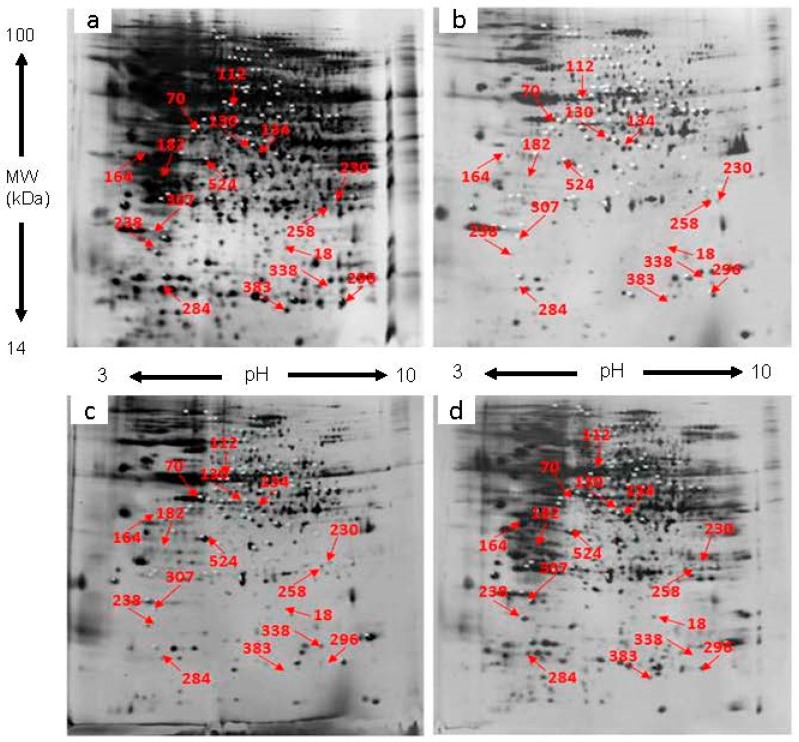
Representative 2-DE gel images of pistil protein profiles of *D. chrysanthum*. (**a**,**b**) 2-DE gel images of SP pistils protein profiles at 2 h and 4 h post-pollination, left and right images, respectively; (**c**,**d**) 2-DE gel images of CP pistils protein profiles at 2 h and 4 h post-pollination, left and right images, respectively. Some of the selected protein spots are demonstrated with red arrows.

Approximately, 1500 protein spots were reproducibly detected in each gel (Figure 1 and Figure 2). In addition, these proteins were distributed evenly in the range of molecular mass 19–97 kDa and pI value 4–9. Out of the 1500 protein spots, there are 1126 common expressed protein spots in SP vs. un-pollination, 1431 in CP vs. un-pollination at 2 h, respectively;there are 1154 common expressed protein spots in SP vs. un-pollination, 1147 in CP vs. un-pollination at 4 h, respectively. While there were 374 differentially expressed protein spots in SP vs. un-pollination, 69 in CP vs. un-pollination at 2 h, respectively; there were 346 differentially expressed protein spots in SP vs. un-pollination, 353 in CP vs. un-pollination at 4 h, respectively. These differentially expressed proteins might be independently or cooperatively involved in the regulation of pollination.

### 2.2. Identification of the DEP

These protein spots corresponding to the differentially expressed proteins (DEPs) from the SP and the CP pistils were selected and excised from the 2-DE gels, and analyzed by matrix-assisted laser desorption/ionization source and tandem time-of-flight (MALDI-TOF/TOF) mass spectrometry. The mass spectrometry (MS) data was searched against NCBI protein database by using the Mascot search engine. A total of 54 proteins were successfully analyzed and identified (Table 1), including accession number, identified protein, Mascot score sequence coverage and function.

Of the 54 protein spots corresponding to DEPs, 13 were more abundant in the self-pollination 2 (SP2), and 40 were more abundant in the self-pollination 4 (SP4) (Table 1). Among the 13 DEPs in the SP2 pistil, the DEP (spot 258, accession number HS521951) and DEP (spot 284, accession number HO192248) were observed abundant. On the other hand, 23 and 27 DEPs were more abundant in the cross-pollination 2(CP2) andcross-pollination 4(CP4) samples, respectively (Table 1). Some DEPs (spot 230, accession number GE489969; and spot 238, accession number HO189451), were more abundant in the CP samples.

### 2.3. Gene Ontology Analysis of Differentially Expressed Proteins between Self-Pollination and Cross-Pollination

**Figure 3:**
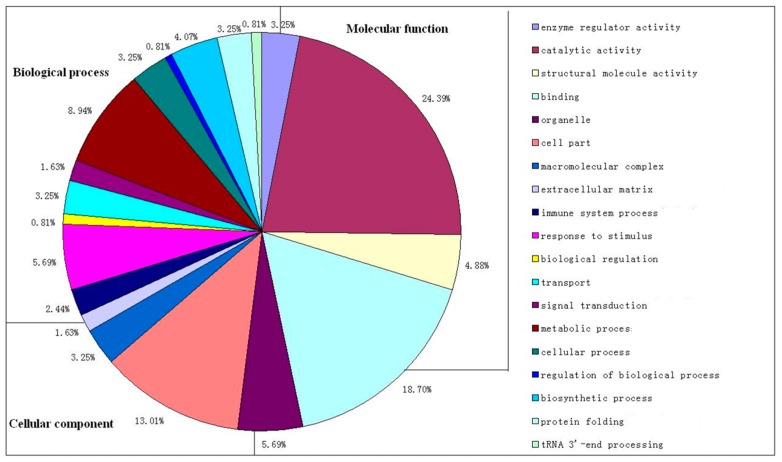
Functional categorization of the identified DEPs between CP and SP through gene ontology (GO) in three domains: cellular component, molecular function, and biological process according to the GO terms.

To gain functional information about these identified proteins, the Basic Local Alignment Search Tool (BLAST) was used to search for homologous proteins against the National Centre for Biotechnology Information (NCBI) non-redundant protein database. The summarized Gene Ontology (GO) mapping and annotation data of the DEPs between self-pollination and cross-pollination were shown in Figure 3. Enrichment analysis against agriGO showed that the identified proteins were associated with a wide variety of cellular processes. They were classified into the following categories according to their function, including metabolic process (8.94%), response to stimulus (5.69%), biosynthetic process (4.07%), protein folding (3.25%) and transport (3.25%). Some proteins were involved in other biological functions, such as catalytic activity, binding, cell structure, and immune. These protein groups are likely to have critical roles in the early response of pollination. Other studies also revealed that protein biosynthesis, stress response, and metabolic process related proteins were involved in the reactions of pollen-stigma recognition in soybean [34]. Also, study on *Brassica napus* found that biosynthesis, signal transduction, cytoskeleton, and exocytosis related proteins were significantly changed between SI and CI, indicating that these kinds of proteins play crucial rules in the early stage of pollinationin a vary of species [35]. These differentially expressed proteins in our study will provide valuable information to investigate the mechanisms concerning the early response before the pollen tube elongation occurring in orchids. We also found that some proteins associated with immune system were significantly changed between the SP and CP samples in this study, suggesting that there is probably an orchids-specific mechanism controlling the early pollination response and possibly contribute to the later GSI,. These proteins will provide a new idea for the study of the complex regulation mechanism of self-incompatibility in orchids.

### 2.4. Functional Categories of the DEGs

To reveal common or different features between the biological characteristics of the SP and CP pistils, the functional categories of the DEPs and the proportion in each category of the SP and CP pistils were compared. The spot number profiles for the functional categories of these two sets of proteins and the relative expression levels of the proteins in each of these functional categories were analyzed (Figure 3). The functional categories of stigma related proteins in SP and CP pistils were found to contain the highest numbers of protein spots. Notably 9% of the DEPs were assigned to the ‘metabolic process’ category, especially those involved in energy metabolism pathways including glycolysis, tricarboxylic acid cycle, pentose phosphate pathway, oxidative phosphorylation, and fatty acid metabolism.

According to the presence or absence of surface exudates, stigmas are generally classified into wet and dry categories. In addition, wet stigmas secrete the liquid exudates containing proteins, liquids, carbohydrates, and water to their surface, which has been shown to be necessary for pollen-stigma interactions during pollination. Recently, specifically and preferentially expressed proteins in wet and dry stigmas of Arabidopsis (*Arabidopsis thaliana*), maize (*Zea mays*), tobacco (*Nicotianatabacum*) and rice (*Oryza sativa*) were identified [36]. We found that *D. chrysanthum* is a wet stigma plant species [37,38]. One protein, HO192673 (spot 18), was found to be more highly expressed in the cross-pollination pistil. This protein was predicted to function in fatty acid β-oxidation. Previous studies revealed that fatty acid β-oxidation related proteins were involved in the growing pollen tube, and also showed that these proteins could be one of the components of the wet stigma surface exudates [36,39]. In this study, we observed the differential expression of fatty acid β-oxidation (spot 18) at the early stage of pollination, indicating that the fatty acid β-oxidation proteins were responsible for the recognition of pollination and may be also involved in sporophytic mechanisms.

In addition, some amino acids metabolism-related enzymes, such as NP_001048045 (spot 182), XP_002319710 (spot 112) were identified to be decreased at 2 h in the SP pistil. These proteins are important enzymes involved in the reaction converting glutamate to glutamine, which help assimilate ammonia into glutamine for its transport in plants [40,41,42,43,44]. It was also reported that glutamate is the precursor for the biosynthesis of γ-aminobutyrate, which plays a critical role in regulating pollen tube growth [45,46].

Furthermore, some other proteins associated with oxidative phosphorylation, such as CAN70186 (spot 134), EMJ11768 (spot 139), also had higher expression levels at 2 h in the cross-pollination pistil. Research showed than NADPH was thought as SI related protein [47], so these proteins may act to maintain higher respiration rate to meet the demands for ATP in SI responses [48]. In addition, this kind of DEP exhibited in the early pollination response also indicated that the differential reaction referring to respiration demands for ATP may take place when pollen-stigma interacted.

The second largest group of the differentially expressed proteins in self-pollinations and cross-pollinations belong to those involved in response stimulus. Recent studies in rice and tobacco have shown that the stress/defense and pollination response pathways were composed of similar gene sets, suggesting cross-talks between pollination and stress responses [49,50,51,52,53]. Some defense-related proteins were identified in our current analysis, such as HO849917 (spot 383) and HO198066 (spot 388). These proteins may respond to pollination and play key roles in the interactions between pollen grains and the stigma.

Moreover, stress may induce the accumulation of reactive oxygen species (ROS) in plants, which can cause damage to plant cells and trigger stress responses [54]. Constitutive presence of ROS is a feature of angiosperm stigma [55]. Because ROS could either be a secondary messenger or be a toxic molecule, determined by its concentration, plants initiate a ROS homeostasis system to keep ROS in an endurable concentration during pollination. In our study, protein ACJ38541 (spot 164) and ACJ38537 (spot 307), belonging to the Pyr-redox protein, were found to be increased at the cross-pollination pistil which indicated that plants invoke the glutathione–ascorbate cycle pathway to reduce excess ROS in self-pollination [56,57].

In our dataset, we found the expression of some ubiquitin-related proteins was dramatically increased at the cross-pollination pistil, including HO189346 (spot 296) at 4 h after pollination, and NP_179435 (spot 70) at 2 h after pollination. Ubiquitin acts as degradation signals for proteolysis and recent studies have shown that SI responses might be activated by a phosphorylation-mediated ubiquitination mechanism after pollen grains land on pistil [58,59]. These data provided proteomic evident that the ubiquitination mechanism which are key regulators in the *S-RNase* based GSI [21], may also play a crucial role at the early stage of pollination response. We also identified the translational elongation factor EF-TuM (spot 524) as an up-regulated protein in cross-pollination. EF-TuM is a microtubule-associated protein and binds to the microtubule lattice [60,61,62,63], which can modulate microtubule dynamics in vitro and in vivo [64,65,66,67,68,69]. The role of EF-TuM in pollination might be related to the production and elongation of pollen tubes. However, at the early stage of pollination the pollen growth has not occurred. Therefore, the EF-TuM is likely to be involved in sporophytic mechanism, and we can infer that the stigma microtubule may also have a crucial role in the early pollination response. In addition, we found the expression of phosphopyruvate hydratase activity-related protein CB033636 (spot 31) was increased in SP4, CP2 and CP4 samples, but decreased in the SP2 sample. The phosphopyruvate hydratase was reported to be involved in the defense against fungi [70,71]. Previous studies have shown that a molecule associated with powdery mildew resistance, NORTIA (NTA), plays a role in the process of reception pollen tubes in synergids, suggesting that this protein is involved in both pollen tube acceptance and powdery mildew infections [72] and the decrease in SP2 may contribute to pollination response at early stage. We also found the expression of transferase activity-related protein HO193941 (spot 33) was increased in CP but decreased in SP, suggesting a positive function for the CP in this process. ATP is not only a major source of biochemical energy for living cells, but also acts as a signaling molecule through inter-cellular communication [73,74]. Some proteins associated with ATP-binding, such as CK857713 (spot 91), HS521850 (spot 151) and HO197113 (spot 184), were detected to be differentially expressed between SP and CP samples, suggestingthat the energy metabolism is highly related to the early pollination response.

In conclusion, our proteomic analysis revealed several important classes of proteins that were differentially expressed in self-pollination and cross-pollination. The putative functions of these proteins indicate that the enhancement of primary metabolism, expression of stress-related proteins, and biosynthesis of microtube compounds might be the key factors contributing to successful pollination. We hope these findings will further our understanding of orchid reproduction and particularly for uncovering the molecular mechanisms underlying pollination response in orchids.

## 3. Materials and Methods

### 3.1. Plant Materials

The plants of *D. chrysanthum* (Plant specimen number: Z.J.Liu3606) were grown under natural conditions in the Orchid Conservation & Research Center of Shenzhen, south of China. The buds of *D. chrysanthum* were emasculated one day before flowering, and artificially pollinated on the day after flowering. Un-pollinated pistils were used as negative controls. Three groups of samples were collected from un-pollinated, self-pollinated and cross-pollinated plants, respectively. Pistil samples were collected at 2 h and 4 h after pollination. The collected samples were immediately frozen in liquid nitrogen and stored at −80 °C for protein extraction.

### 3.2. Protein Extraction and Quantification

Pollinated pistils (approximately 100 mg) frozen in liquid were ground using pestle and mortar and then suspended in 1.5 mL extraction solution (10% *w/v* TCA in acetone, 1 mM PMSF and 0.2% DTT) for 2 h. The mixtures were then centrifugated for 20 min at 12,000× *g*, and the pellets were washed with prechilled acetone containing 0.2% DTT. A following centrifugation at 12,000× *g* for 20 min was performed, and the pellets were dried under vacuum for 30 min. The dried protein pellets were dissolved in lysis buffer containing 7 M urea, 2 M thiourea, 4% (*w/v*) CHAPS, 65 mM DTT, 2% (*v/v*) IPG, 10 mM PMSF and incubated at 25 °C for 2 h. After centrifuging at 12,000× *g* for 20 min, the supernatants were collected for protein quantification. Protein concentration was measured by the Bradford method with Bovine serum albumin (BSA) as a standard. Three biological replicates were used, and one biological replicate was a mixture of combined plant samples derived from the same treatment.

### 3.3. Two-Dimensional Gel Electrophoresis

Two-dimensional gel electrophoresis was performed according to the protocol by Shen et al. with minor modifications [75]. For the first-dimension IEF, the IPG strips were rehydrated in 250 μL rehydration buffer (8 M urea, 2% (*w/v*) CHAPS, 0.5% (*v/v*) Pharmalyte pH 3–10, and 0.002% bromophenol blue) containing 150 μg protein sample. Precast 13 cm immobilized pH gradient (IPG) strips (non-linear pH = 3–10; GE Healthcare, Little Chalfont, UK) were rehydrated for 12 h at 30 V. IEF conditions were performed with the following voltage program: 100 V/2 h, 200 V/1 h, 500 V/1 h, linear ramp to 1000 V over 1 h, 8000 V over 3 h, then 8000 V constant for a total focusing time of 55,000 Vh. After IEF, IPG strips were incubated with 10 mM DNPH in 2 M HCl for 10 min at room temperature and washed with 2 M Tris/30% glycerol (*v/v*) for 15 min, and then incubated for 15 min in equilibration buffer consisting of 6 M urea, 30% (*v/v*) glycerol, 2% (*w/v*) SDS, 2% (*w/v*) DTT and 0.05 M Tris–HCl, pH 6.8, and subsequently for 15 min in the same buffer containing 2.5% (*w/v*) iodoacetamide instead of DTT. The second dimensional SDS-PAGE was performed on 12% polyacrylamide gels using SE 600 Ruby system (GE healthcare). The gels were first fixed in 50% MeOH, 12% HAc and 0.05% formalin for 2 h. The SDS-PAGE was first run at a current of 10 mA/gel for 30 min and then at a constant current of 20 mA/gel at 15 °C until bromophenol blue reached the gel bottom. Then the gels were stained in 0.2% AgNO_3_ and 0.076% formalin for 20 min. Finally, gels were developed with 6% Na_2_CO_3_, 0.05% formalin and 0.0004% Na_2_S_2_O_3_. Staining was stopped with 50% MeOH and 12% HAc for 5 min. 2-DEs were performed for each biological replicate individually, and the result of each treatment was the mean of three biological replicates.

### 3.4. Image Acquisition and Data Analysis

The silver-stained gels were scanned using the proXPRESS 2D imaging system (PerkinElmer, Hong Kong, China). The images were analyzed with ImageMaster 2D Platinum software version 5.0 (GE Healthcare). All 2-DE images were analyzed by the software and the identified spots were manually rechecked. The experiments were repeated two times for each sample. Only those spots that showed significantly different were considered to be differentially expressed proteins (DEPs) and characterized by mass spectrometry (MS) (*p* < 0.05). The un-pollination sample was used as control, and the DEP was the comparison between SP and CP.

### 3.5. Protein Identification with MALDI-TOF/TOF MS

For protein identification, the DEP spots of interest were excised from the 2-DE gels and destained for 30 min. Thentryptic in-gel digestion was performed [75]. Gel chips were destained in a 1:1 solution of 30 mM potassium ferricyanide and 100 mM sodium thiosulfate and then equilibrated in 50 mM ammonium bicarbonate to pH 8.0. After hydrating with 100% acetonitrile (ACN) and drying in a Speed Vac, the gel slices were rehydrated in a minimal volume of trypsin solution (10 μg/mL trypsin, 25 mM NH_4_HCO_3_) and incubated at 37 °C for 16 h.

After trypsin digestion, the protein peptides were collected for matrix-assisted laser desorption/ionization time of flight mass spectrometry (MALDI-TOF–MS) analysis on a 5800 MALDI TOF/MS mass spectrometry (AB SIEX, Framingham, MA, USA). Protein digestion extracts (tryptic peptides) were resuspended with 5 μL of 0.1% trifluoroacetic acid and then the peptide samples were mixed (1:1 ratio) with a matrix consisting of a saturated solution α-cyano-4-hydroxy-trans-cinnamic acid in 50% acetonitrile, 1% trifluoroacetic acid. 0.8 µL liquots were spotted onto stainless steel sample target plates.

Peptide mass spectra were acquired in positive ion reflection mode, and 800–4000 *m/z* mass range with 1000 laser shots was used. Precursor ions were selected for MS/MS analysis according to fixed criteria (20 most intensive peaks, S/N > 50). Energy of 1 kV was used for collision-induced dissociation (CID), air was used as collision gas and 2000 acquisitions were accumulated for each MS/MS spectrum. Combined MS and MS/MS spectra were searched in the Swiss-Prot database with peptide mass fingerprinting using Mascot 2.3.02. The search was performed with the following parameters: trypsin as proteolytic enzyme with only one missed cleavage site was accepted; 100 ppm for precursor ion tolerance and 0.3 Da for fragment ion tolerance. The identification process was repeated three times using appropriate protein spots from three different silver-stained gels. To determine the confidence of the identification proteins, three rules were applied. First, probability-based MOWSE *p* < 0.05; Second, the identified proteins have to match at least 5 peptides and more than 10% protein sequences coverage; Third, when matched peptides have multiple homologous proteins, only the peptide with the highest confidence were selected.

### 3.6. Statistical Analysis and Gene Ontology Analysis

All data are presented as mean ± SD and statistical analyses were performed by the two-tailed Student’s *t*-test. Protein spots with fold change > 2 and *p* < 0.05 were considered as significant differentially expressed. The molecular function, cellular components and biological process of the differentially expressed proteins (DEPs) were analyzed according to the Gene Ontology (GO) database (http://www.geneontology.org/). GO enrichment analysis provides all GO terms which are significantly changed in the CP when compared with the SP.

## Figures and Tables

**Table 1:** Differentially accumulated proteins identified by MS. Protein spot number refers to numbers in Figure 1 and Figure 2. Accession number and Protein name according to the best hit of MASCOT search against NCBInr database and plant EST database. Functional protein classification according to the Uniprot database. Fold increase and decrease were calculated as SP (CP)/control and—control/SP (CP) for up and down-regulated proteins respectively. All fold changes shown are statistically significant (*p* < 0.05). PI isoelectric points, MW molecular weight, SP self-pollination, CP cross-pollination.

Spot Number	Protein Name	Accession Number	MW (Da)	Protein PI	Mascot Score	Matched Peptides	Sequence Coverage (%)	Function	Fold Increase (+) or Decrease (−)			
									SP 2	SP 4	CP 2	CP 4
18	Dendrobiumnobile cDNA	HO192673	27,176	8.31	408	3	20	fatty acid β-oxidation	−31.2 ± 2.23	−1.8 ± 0.14	31.2 ± 2.46	20.8 ± 1.22
31	Phalaenopsisequestris cDNA clone EFCP035A12	CB033636	24,814	9.1	235	2	15	Phosphopyruvatehydratase activity	−34.4 ± 1.80	8.6 ± 0.50	15.8 ± 0.87	34.4 ± 3.04
33	Dendrobiumnobile cDNA	HO193941	27,570	6.03	194	2	12	Transferase	−7.6 ± 0.59	−8.4 ± 0.47	4.8 ± 0.43	8.4 ± 0.63
62	Dendrobiumnobile cDNA	HO189262	29,674	5.81	368	3	19	response to stress	−16.3 ± 1.51	16.3 ± 1.15	9.5 ± 0.77	−1.9 ± 0.17
70	flavoprotein subunit 2 [Arabidopsis thaliana]	NP_179435	70,015	5.85	189	4	9	mitochondrial electron transport, succinate to ubiquinone	−16.1 ± 1.40	10.4 ± 0.90	16.1 ± 1.39	8.7 ± 0.61
71	Texas blueweed Helianthus ciliaris CHCL8946	EL420682	31,916	5.93	80	1	5	Zein-binding	−19.4 ± 1.03	19.4 ± 1.02	9.2 ± 0.60	5.1 ± 0.35
81	PRUPE_ppa003377mg [Prunuspersica]	EMJ16224	62,014	5.8	179	2	5	metabolic process magnesium ion binding	−6.5 ± 0.59	6.5 ± 0.33	3.0 ± 0.28	−1.8 ± 0.14
91	Phalaenopsisviolacea cDNA	CK857713	29,392	6.99	114	1	6	ATP-binding, protein folding	−20.4 ± 1.24	18.3 ± 1.53	14.3 ± 1.33	20.4 ± 1.17
100	Triticum aestivum cDNA clone wl1n.pk0005.g10	CA616775	17,053	9.3	82	1	9	transmembrane transport	−29.7 ± 2.22	13.3 ± 1.15	13.1 ± 0.74	29.7 ± 2.44
107	Festuca pratensis cDNA clone 29N21	GO893814	23,943	10.05	76	1	6	uncharacterized protein	−46.4 ± 3.34	13.6 ± 0.76	37.0 ± 2.83	46.4 ± 2.99
112	5-methyltetrahydropteroyl triglutamate-homocysteine [Populustrichocarpa]	XP_002319710	85,370	6.1	261	4	6	zinc ion binding, methionine biosynthetic process	−91.9 ± 6.03	11.3 ± 0.96	91.9 ± 6.36	48.6 ± 3.53
127	monodehydroascorbate reductase [Oncidium hybrid cultivar]	ACJ38541	46,809	5.26	205	4	17	oxidoreductase activity	−80.5 ± 6.31	19.3 ± 1.48	6.7 ± 0.58	−3.5 ± 0.22
128	Malus x domestica cDNA	DT002244	24,691	8.31	73	1	6	Acyltransferase	−215.2 ± 11.83	71.8 ± 3.97	215.2 ± 16.77	5.9 ± 0.41
134	VITISV_034728 [Vitis vinifera]	CAN70186	53,150	6.76	280	5	12	generating NADPH	−4.5 ± 0.29	2.0 ± 0.13	4.5 ± 0.32	2.7 ± 0.16
139	PRUPE_ppa003869mg [Prunuspersica]	EMJ11768	59,411	6.69	116	2	4	oxidoreductase activity NAD or NADP as acceptor	−12.7 ± 0.75	4.2 ± 0.28	10.0 ± 0.91	12.7 ± 1.31
151	Oncidium Gower Ramsey cDNA	HS521850	30,518	6	166	2	11	ATP-binding, Metal-binding, succinate-CoA ligase activity	−1.1 ± 0.10	2.9 ± 0.27	2.0 ± 0.17	2.8 ± 0.24
154	Soybean Seeds Containing Globular-Stage Embryos Glycine max cDNA	GD856994	3073	5.69	73	1	55	protein methyltransferase activity	−15.8 ± 1.42	12.8 ± 0.94	15.8 ± 1.12	14.8 ± 1.34
161	Phalaenopsis equestris cDNA clone EFCP035E06	CB033673	20,026	7.83	390	3	32	magnesium ion binding Methionine biosynthesis	−10.4 ± 0.62	3.0 ± 0.18	10.4 ± 0.78	2.0 ± 0.16
162	DAFB seeds Malus x domestica cDNA clone AAWA002059	CN887431	21,889	11.14	70	1	5	May play a role in plant defense	−15.2 ± 0.88	5.6 ± 0.30	10.3 ± 0.81	15.2 ± 0.88
164	monodehydroascorbate reductase [Oncidium hybrid cultivar]	ACJ38541	46,809	5.26	206	4	17	oxidoreductase activity	−343.3 ± 20.59	47.3 ± 3.42	136.3 ± 7.43	343.3 ± 25.51
165	Dendrobiumnobile cDNA	HO189275	26,298	7.88	419	4	22	phosphoglycerate kinase activity	−13.3 ± 0.72	13.3 ± 1.01	5.2 ± 0.39	2.3 ± 0.17
178	Triphysariaversicolor cDNA	EY010367	23,141	10	77	1	6	hydrolase activity	−20.5 ± 1.55	2.8 ± 0.17	11.7 ± 0.94	20.5 ± 1.38
182	Os02g0735200 [Oryza sativa Japonica Group]	NP_001048045	39,405	5.51	238	2	14	High-affinity glutamine synthetase	−35.8 ± 3.07	35.8 ± 2.55	21.0 ± 2.09	20.6 ± 1.62
184	Dendrobiumnobile cDNA	HO197113	25,515	5.7	176	1	12	ATP binding, MAP kinase activity	−25.1 ± 1.53	9.9 ± 0.84	7.2 ± 0.40	25.1 ± 2.49
187	Solanumhabrochaites cDNA	GT169059	40,752	8.53	246	3	11	GTP-binding, protein transport	−10.1 ± 0.82	1.2 ± 0.09	5.9 ± 0.31	10.1 ± 0.89
204	Dendrobiumnobile cDNA	HO203854	30,487	8.35	221	2	13	tricarboxylic acid cycle malate metabolic process	−1.1 ± 0.10	1.8 ± 0.14	2.2 ± 0.16	3.1 ± 0.23
	Dendrobiumnobile cDNA	HO196589	23,411	7.82	161	2	12					
217	Dendrobiumnobile cDNA	HO203393	29,572	9	234	3	16	malate metabolic process Oxidoreductase	−14.5 ± 0.81	14.5 ± 0.85	10.2 ± 0.75	14.0 ± 0.83
221	peptide ABC transporter substrate -binding protein [Bacilluscereus]	YP_002368400	63,740	8.69	662	5	13	Signal, ATP-driven transport Metal-binding	−2.2 ± 0.21	2.2 ± 0.15	3.9 ± 0.29	3.9 ± 0.23
230	sunflower Helianthus annuus cDNA clone CCFS4413	GE489969	30,766	9.36	86	1	5	microtubule motor activity	2.5 ± 0.21	10.8 ± 0.90	9.0 ± 0.51	3.8 ± 0.24
232	Vanda hybrid cultivar cDNA	GW392872	19,682	9.47	119	1	9	oxidoreductaseactivity, zinc ion binding	−4.6 ± 0.26	4.8 ± 0.31	3.2 ± 0.27	4.8 ± 0.28
233	Dendrobiumnobile cDNA	HO195954	27,748	5.56	112	1	7	carboxylesterase activity	−13.6 ± 1.19	10.0 ± 0.93	13.1 ± 0.90	2.9 ± 0.21
238	Dendrobiumnobile cDNA	HO189451	25,061	5.47	217	2	13	Unknown protein	−14.7 ± 1.46	8.4 ± 0.51	14.7 ± 0.10	13.2 ± 0.94
240	Mimulusguttatus cDNA clone CCIG14980	GR000041	27,311	9.2	109	1	6	regulation of translational initiation, translation initiation factor activity	2.4 ± 0.19	11.0 ± 0.84	12.2 ± 0.77	6.7 ± 0.40
247	putative enoyl-ACP-reductase protein [Elaeisguineensis]	AEZ00840	38,749	9.27	229	3	18	Oxidoreductase, enoyl-[acyl-carrier-protein] reductase (NADH) activity	2.7 ± 1.67	5.0 ± 0.41	4.5 ± 0.35	5.0 ± 0.33
249	Dendrobiumnobile cDNA	HO192097	24,344	5.22	152	2	11	response to stress, oxidoreductase activity	4.4 ± 0.36	6.2 ± 0.45	8.2 ± 0.64	13.7 ± 1.21
	Dendrobiumnobile cDNA	HO190191	26,789	9.13	156	2	11					
258	Oncidium Gower Ramsey cDNA	HS521951	30,535	8.96	194	2	11	Uncharacterized protein	1.4 ± 0.09	4.0 ± 0.20	2.9 ± 0.27	6.1 ± 0.41
261	Dendrobiumnobile cDNA	HO196032	28,575	5.99	252	2	11	Uncharacterized protein	3.1 ± 0.25	2.2 ± 0.20	4.5 ± 0.23	4.5 ± 0.27
	Dendrobiumnobile cDNA	HO201509	27,739	8.26	318	2	14					
284	Dendrobiumnobile cDNA	HO192248	23,859	5.75	99	1	6	Uncharacterized protein	2.3 ± 0.21	−3.1 ± 0.30	3.1 ± 0.22	5.2 ± 0.30
294	Panicumvirgatum cDNA	JG964858	30,004	9.4	84	1	5	transporter activity	2.0 ± 0.17	2.7 ± 0.22	4.6 ± 0.38	4.6 ± 0.44
296	Dendrobiumnobile cDNA	HO189346	23,335	9.36	271	2	18	ubiquitin-dependent protein	3.6 ± 0.32	−9.2 ± 0.85	12.3 ± 1.16	12.3 ± 0.91
303	fibrillin-like protein [Oncidium hybrid cultivar]	AAY24688	34,734	5.48	85	2	10	structural molecule activity	1.4 ± 0.10	2.2 ± 0.14	4.2 ± 0.40	4.2 ± 0.32
	Oncidium hybrid cultivar cDNA	HS524185	24,403	8.12	249	2	14					
307	ascorbate peroxidase [Oncidium hybrid cultivar]	ACJ38537	27,441	5.34	241	2	20	response to oxidative stress	1.2 ± 0.11	2.8 ± 0.22	4.1 ± 0.28	4.1 ± 0.32
311	Dendrobiumnobile cDNA	HO202862	29,065	5.26	655	4	26	triose-phosphate isomerase activity	1.3 ± 0.12	3.1 ± 0.21	3.7 ± 0.29	3.7 ± 0.22
320	Oncidium Gower Ramsey cDNA	HS522419	32,789	5.48	79	1	4	Zinc phosphodiesterase, Endonuclease	−1.5 ± 0.12	2.3 ± 0.19	2.2 ± 0.20	1.9 ± 0.14
329	Coffeaarabica cDNA clone CAET42MIX-CFEZE47TVC	GT010034	32,267	8.06	139	1	5	Thaumatin-like protein	2.1 ± 0.18	8.7 ± 0.85	3.7 ± 0.25	8.1 ± 0.70
357	Dendrobiumnobile cDNA	HO198288	24,617	7.72	217	3	15	PPIases accelerate the folding of proteins	−15.3 ± 0.91	10.3 ± 0.84	15.3 ± 1.08	12.4 ± 1.09
360	unknown [Piceasitchensis]	gi|116779193	18,169	8.34	102	2	14	peptidyl-prolylcis-trans isomerase activity	−11.0 ± 0.77	11.0 ± 0.83	6.0 ± 0.51	−4.4 ± 0.24
363	peroxiredoxin 5 cell rescue protein [Loliumperenne]	AFA36612	11,445	5.13	160	2	31	oxidation-reduction process	2.0 ± 1.48	2.5 ± 0.19	6.6 ± 0.34	6.6 ± 0.62
383	Ophrysfusca cDNA clone Ofup2722	HO849917	19,693	8.11	98	1	7	defense response	−9.6 ± 0.91	5.6 ± 0.31	−14.7 ± 1.06	14.7 ± 0.79
388	Dendrobiumnobile cDNA	HO198066	24,490	9.24	235	3	17	defense response	−17.9 ± 1.28	11.4 ± 0.88	5.6 ± 0.54	18.0 ± 1.47
464	lettuce serriola Lactuca serriola cDNA clone QGH6B22	BU007993	23,103	4.84	112	1	9	ATP-binding, Formation of phosphoenolpyruvate	−61.9 ± 3.71	61.9 ± 4.49	50.6 ± 4.57	−1.1 ± 0.10
524	translational elongation factor EF-TuM [Zea mays]	AAG32661	48,746	5.99	390	4	22	translation elongation factor activity, GTP catabolic process	−3.1 ± 0.21	3.1 ± 0.25	2.9 ± 1.74	1.5 ± 0.15
530	M569_05826, partial [Genliseaaurea]	EPS68937	48,363	8.25	291	3	13	transferase activity	−15.0 ± 0.94	15.0 ± 1.24	15.0 ± 1.18	1.6 ± 0.16
531	Dendrobiumnobile cDNA	HO193012	26,029	6.5	299	2	16	zinc-containing alcohol dehydrogenase family	−4.7 ± 0.35	12.2 ± 0.70	9.8 ± 0.60	1.1 ± 0.08
